# [Corrigendum] CLEC4M overexpression inhibits progression and is associated with a favorable prognosis in hepatocellular carcinoma

**DOI:** 10.3892/mmr.2026.14003

**Published:** 2026-09-01

**Authors:** Qianle Yu, Kai Gao

Mol Med Rep 22: 2245–2252, 2020; DOI: 10.3892/mmr.2020.11336

Following the publication of the above article, the authors have contacted the Editorial Office to explain that certain of the western blots featured in Figs. 3A and B, and 6A and B, on p. 2248 and 2250 respectively were chosen incorrectly. Specifically, the data chosen for the CLEC4M blots in Fig. 3A and B, and the p-JAK1 blots in Fig. 6A and the STAT3 blots in Fig. 6B, were presented incorrectly in these figures.

However, the authors retained their original data, and have submitted corrected versions of Figs. 3 and 6, now showing the correct data for the CLEC4M blots in Fig. 3A and B, and the p-JAK1 blots in Fig. 6A and the STAT3 blots in Fig. 6B. These figures are featured on the next page. Note that the errors made in compiling these figures did not affect the results or the main conclusions reported in the study. All the authors approve of the publication of this corrigendum, and are grateful to the Editor of *Molecular Medicine Reports* for allowing them the opportunity to publish this. The authors regret their oversight in allowing these errors to be included in the paper, and apologize to the readership for any inconvenience caused.

## Figures and Tables

**Figure 3.**
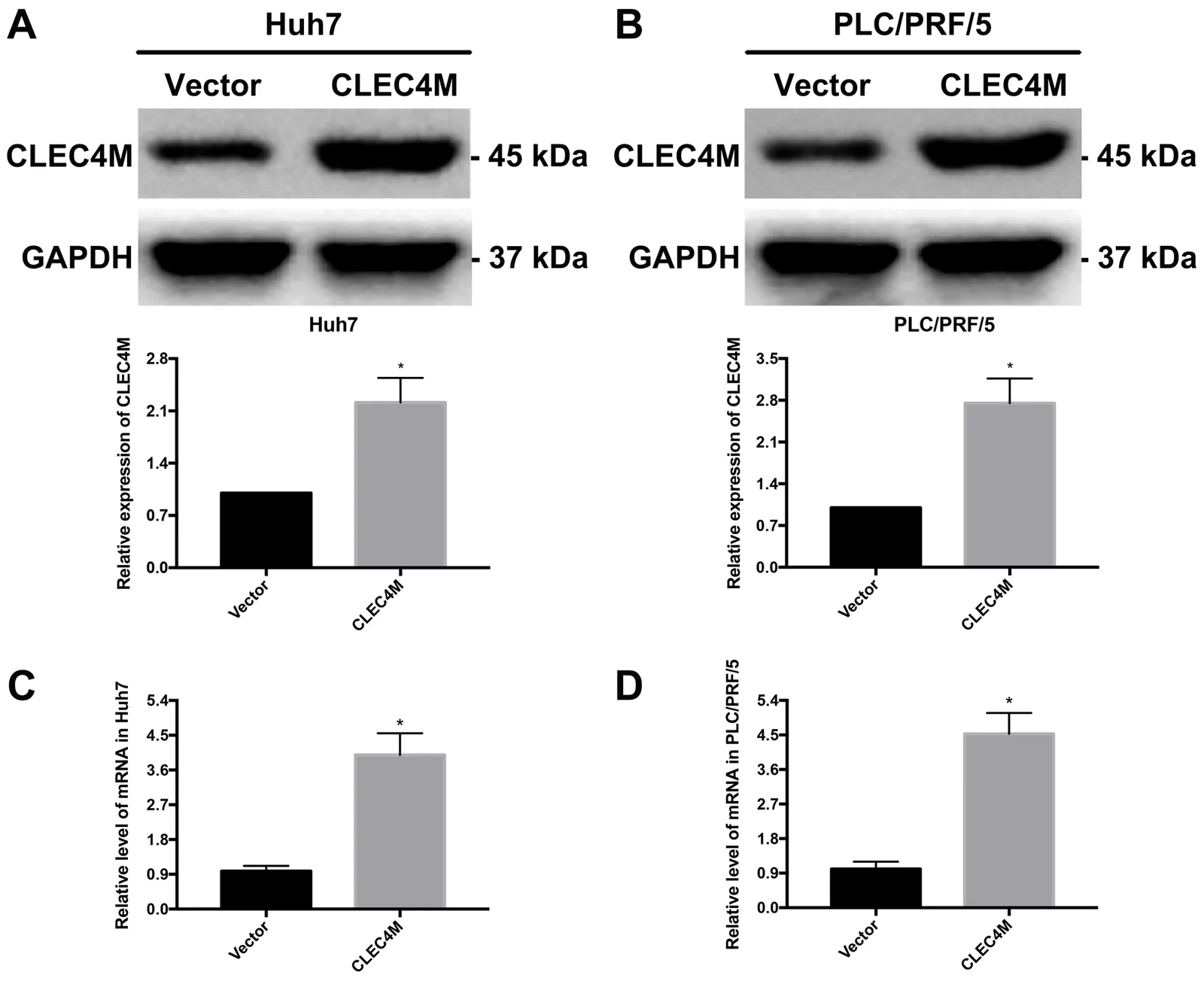
Construction of HCC cell lines with stable overexpression of CLEC4M. CLEC4M protein levels were assessed in infected (A) Huh7 and (B) PLC/PRF/5 cells using western blotting. CLEC4M mRNA levels were assessed in infected (C) Huh7 and (D) PLC/PRF/5 cells via reverse transcription-quantitative PCR analysis. Data are presented as the mean ± standard deviation. *P<0.05. HCC, hepatocellular carcinoma; CLEC4M, C-type lectin domain family 4 member M; Vector, negative control.

**Figure 6.**
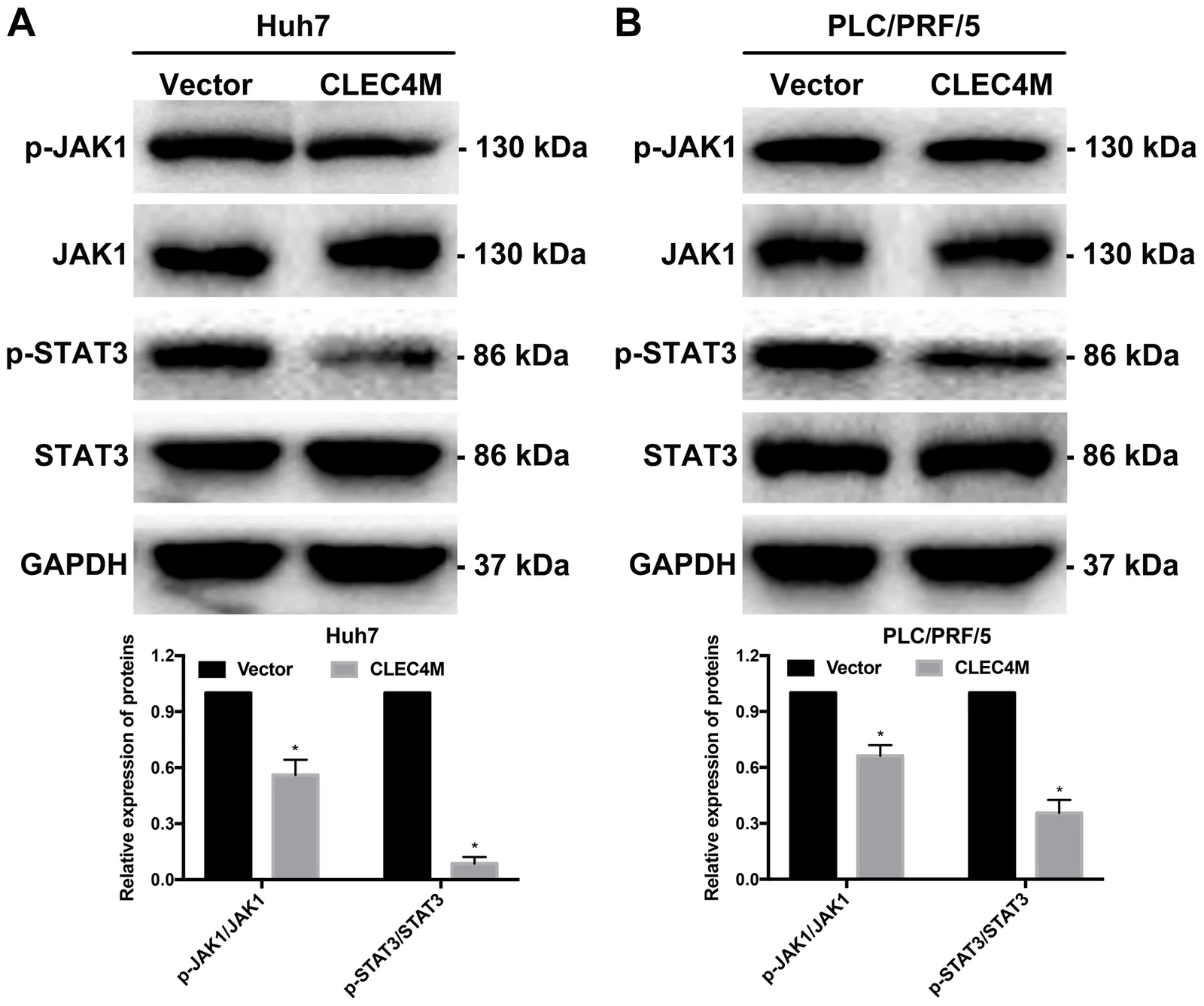
CLEC4M overexpression inhibits the JAK1/STAT3 pathway. p-JAK1, JAK1, p-STAT3, STAT3 and GAPDH protein levels were assessed in infected (A) Huh7 and (B) PLC/PRF/5 via western blotting. Data are presented as the mean ± standard deviation. *P<0.05. CLEC4M, C-type lectin domain family 4 member M; p-, phosphorylated; JAK1, Janus kinase 1; STAT3, signal transducer and activator of transcription 3; Vector, negative control..

